# Nonadherence in diabetes care among US adults with diabetes by stroke status

**DOI:** 10.1371/journal.pone.0260778

**Published:** 2021-12-22

**Authors:** Phoebe Tran, Lam Tran, Liem Tran

**Affiliations:** 1 Department of Chronic Disease Epidemiology, Yale University, New Haven, Connecticut, United States of America; 2 Department of Biostatistics, University of Michigan, Ann Arbor, Michigan, United States of America; 3 Department of Geography, University of Tennessee, Knoxville, Tennessee, United States of America; Montclair State University, UNITED STATES

## Abstract

**Objective:**

Effects of stroke (i.e., memory loss, paralysis) may make effective diabetes care difficult which can in turn contribute to additional diabetes related complications and hospitalization. However, little is known about US post-stroke diabetes care levels. This study sought to examine diabetes care levels among US adults with diabetes by stroke status.

**Methods:**

Using 2015–2018 Behavioral Risk Factor Surveillance System surveys, the prevalence of nonadherence with the American Diabetes Association’s diabetes care measures (<1 eye exam annually, <1 foot exam annually, <1 blood glucose check daily, <2 A1C tests annually, no receipt of annual flu vaccination) was ascertained in people with diabetes by stroke status. A separate logistic regression model was run for each diabetes care measure to determine if nonadherence patterns differed by stroke status after adjustment for stroke and diabetes associated factors.

**Results:**

Our study included 72,630 individuals, with 9.8% having had a stroke. Nonadherence levels varied for each diabetes care measure ranging from 20.4–42.2% for stroke survivors and 22.8–44.0% for those who had never had stroke. By stroke status, nonadherence with diabetes management measures was comparable except for stroke survivors having both a lower prevalence (30.2% versus 40.1%) and odds of nonadherence (OR: 0.73, 95% CI: 0.65, 0.82) with daily blood glucose check than those who had never had stroke.

**Conclusion:**

While nonadherence with diabetes management does not vary by stroke status, considerable nonadherence still exists among stroke survivors with diabetes. Additional interventions to improve diabetes care may help to reduce risk of further diabetes complications in this population.

## Introduction

In the US, it is estimated that 27.9% of stroke patients have diabetes [1]. Nonadherence to the American Diabetes Association’s diabetes medical care guidelines following a stroke has been linked to an elevated risk of diabetes complications including kidney disease and nerve damage and the need for further hospitalization [2]. However, effects of stroke such as memory loss and paralysis which affect around 50% and 38% of patients respectively may make it difficult for individuals to properly adhere to these guidelines [3–6].

Although poor diabetes medical care can contribute to increased risk of diabetes related complications, little is known about post-stroke diabetes care adherence levels in the US. Prior US research in this area is not reflective of contemporary diabetes care patterns and only considers a few diabetes care measures [7]. Thus, we compared the prevalence of nonadherence with the ADA’s diabetes care measures among US individuals who have had stroke and those who have never had this neurological condition using nationally representative survey data. We also determined estimates of nonadherence with diabetes care measures by stroke status that were adjusted for stroke and diabetes related factors.

## Methods

### Study data and population

Study data comes from the 2015, 2016, 2017, and 2018 iterations of the Behavioral Risk Factor Surveillance System (BRFSS) surveys [8]. We chose to combine data from these four years in order to obtain sufficient numbers of stroke survivors with diabetes for the analyses and because they are the most current survey years available [8]. Consisting of noninstitutionalized individuals >18 years who live in either the 50 US states, Washington DC., Guam, or Puerto Rico; the BRFSS is a yearly cross-sectional survey that is conducted by the Centers for Disease Control and Prevention (CDC) [8]. Potential BRFSS participants are contacted through either a landline or mobile phone number found in commercial phone listings [8]. Once these individuals agree to participate in the survey, trained BRFSS personnel administer and record participants’ self-reported responses to questions on sociodemographic characteristics, chronic health conditions, and healthcare use [8]. To ensure that results from BRFSS conducted analyses are representative of the general US population, the survey utilizes a combination of oversampling of minority groups and rural residents in some parts of the country as well as survey weighting [8]. As all information that can be used to identify survey participants is removed from the BRFSS, obtaining informed consent and approval from an institutional review board are not needed when using BRFSS survey data [8]. Complete information on the 2015–2018 BRFSS sample background can be found in the accompanying codebooks for these survey years [9–12]. All BRFSS materials are made freely available for public use by the CDC and can be accessed at their website [8].

Our study population is comprised of US individuals >45 years who self-reported having diabetes and whose stroke status was known. We did not include individuals <45 years as the etiology of younger stroke patients differs from the majority of stroke patients who are >45 years as well as the low numbers of young stroke patients in the BRFSS contributing to unstable study estimates [13]. Individuals in the study were identified through the BRFSS diabetes question, “(Ever told) you have diabetes” [13]. In following with other diabetes studies that used BRFSS survey data, people with “Yes” responses to this question were classified as having diabetes while those with “Yes, but female told only during pregnancy”, “No”, “No, pre-diabetes or borderline diabetes”, “Don’t Know/Not Sure”, “Refused”, or “Not asked or Missing” responses (collectively <0.25%) to the diabetes question were excluded from the study [14, 15]. Stroke status information was obtained through the BRFSS stroke question, “(Ever told) you had a stroke” with people considered to have had a stroke if they responded “Yes” to this question, never to have had a stroke if their response was “No”, and excluded from the study if their response was either “Don’t Know/Not Sure”, “Refused”, or “Not asked or Missing” (collectively <0.30%) [12].

### Outcome and covariates

The outcome assessed in this study was nonadherence with the ADA’s diabetes care measures for eye exams, foot exams, blood glucose checks, A1C tests, and flu vaccination [4]. Data on these measures which were only administered to individuals with diabetes was extracted from a series of questions contained in the BRFSS’s diabetes module (“When was the last time you had an eye exam in which the pupils were dilated?”, “About how often do you check your feet for any sores or irritations?”, “About how often do you check your blood for glucose or sugar?”, “About how many times in the past 12 months has a doctor, nurse, or other health professional checked you for “A one C”?”, “During the past 12 months, have you had either a flu shot or a flu vaccine that was sprayed in your nose?”) [12]. Nonadherence for each of these five measures, was based off ADA guidelines with people considered nonadherent if they had <1 eye exam annually, <1 foot exam annually, <1 blood glucose check daily, <2 A1C tests annually, or did not receive a flu vaccination every year [12]. Although one time receipt of pneumonia vaccination and taking a diabetes management course once are part of the ADA’s diabetes care measures, we did not include them in our study since these are not daily/annual measures and a large number of people may have had a pneumonia vaccination and/or completed diabetes care education before their stroke occurred [12]. People with “Don’t Know/Not Sure”, “Refused”, or “Not asked or Missing” responses (collectively <5%) to the diabetes care measures were excluded from the study. Information on stroke and diabetes related sociodemographic characteristics (age, sex, race, education, household income, employment status) that had been identified from existing studies on these conditions was also obtained from the BRFSS [3, 16].

### Statistical analyses

By stroke status, the survey-weighted distribution of sociodemographic characteristics was determined. We also calculated the survey weighted levels of nonadherence with the ADA measures for both people who have and have never had stroke. Adjusted estimates of nonadherence with the ADA measures were assessed through the use of statistical models. A separate survey-weighted logistic regression model that included stroke status, age, sex, race, education, household income, and employment status as covariates was created for each of the five diabetes care measures. In order to account for the influence of missing sociodemographic data on study estimates, indicator variables were used in the models for each sociodemographic factor that had “Refused/Missing” responses [17]. Statistical analyses in the study were conducted using R Version 4.0.

## Results

Our study included 72,630 individuals. Demographic details of the study population, stratified by prior stroke status, are displayed in Table 1. Both sets of study participants were predominantly 55 or older and identified as White. Relative to participants who never had a stroke, those who did were more likely to be 55 or older (85.2%% vs. 79.7%), identify as Black (20.9% vs. 16.4%), completed fewer years of education, have a household income <$50,000 (67.3% vs. 53.7%), and not be in the labor force (83.1% vs. 63.5%).

**Table 1:** Sociodemographic characteristics of 2015–2018 Behavioral Risk Factor Surveillance System study participants with diabetes by stroke status (n = 72630).

Covariates	Had a stroke (n = 7130)		Never had a stroke (n = 65500)		P value
	n	Weighted %^1^ (95% Confidence Interval)	n	Weighted % (95% Confidence Interval)	
**Age**					<0.001
45–54	724	14.8 (14.0, 15.6)	8955	20.3 (20.0, 20.7)	
55–64	1929	31.1 (30.1, 32.2)	19013	32.0 (31.6, 32.3)	
65 or older	4477	54.1 (52.9, 55.2)	37532	47.7 (47.3, 48.1)	
**Sex**					0.842
Male	3159	50.4 (49.2, 51.6)	29586	50.6 (50.3, 51.1)	
Female	3971	49.6 (48.4, 50.8)	35914	49.3 (48.9, 49.7)	
**Race**					<0.001
White	4767	63.0 (61.9, 64.1)	46291	65.4 (65.1, 65.8)	
Black	1303	20.9 (19.8, 21.7)	9465	16.4 (16.1, 16.7)	
Other/Multiracial	526	5.1 (4.6, 5.6)	3892	4.5 (4.4, 4.7)	
Hispanic	412	9.6 (8.9, 10.3)	4780	11.9 (11.7, 12.2)	
Refused/Missing	122	1.5 (1.2, 1.8)	1072	1.7 (1.6, 1.8)	
**Education**					<0.001
Did not complete high school	954	23.0 (22.1, 24.0)	6406	17.6 (17.3, 17.8)	
High school graduate	2432	33.8 (32.7, 34.9)	20760	31.5 (31.1, 31.8)	
Some college or technical school	2179	30.3 (29.3, 31.4)	19004	31.3 (31.0, 31.7)	
College graduate	1554	12.8 (12.0, 13.5)	19177	19.4 (19.1, 19.7)	
Refused/Missing	11	0.1 (0.0, 0.1)	153	0.2 (0.2, 0.3)	
**Household income**					<0.001
Less than $15,000	1417	19.7 (18.8, 20.7)	7710	12.1 (11.8, 12.3)	
$15,000 to <$25,000	1761	25.2 (24.2, 26.2)	12015	18.8 (18.5, 19.1)	
$25,000 to <$35,000	798	11.1 (10.3, 11.8)	6992	10.6 (10.3, 10.8)	
$35,000 to <$50,000	740	11.3 (10.6, 12.0)	8494	12.2 (12.0, 12.5)	
$50,000 or more	1291	18.0 (17.1, 18.9)	20155	31.2 (30.9, 31.6)	
Don’t know/Not Sure/Missing	1123	14.7 (13.8. 15.5)	10134	15.1 (14.8, 15.4)	
**Employment status**					<0.001
Employed	802	12.3 (11.6, 13.1)	18431	31.5 (31.1, 31.8)	
Unemployed	257	4.3 (3.8, 4.8)	2391	4.5 (4.4, 4.7)	
Not in labor force	6046	83.1 (82.2, 84.0)	44410	63.5 (63.2, 63.9)	
Refused/Missing	25	0.3 (0.2, 0.4)	268	0.4 (0.4, 0.5)	

^1^ Survey weighting from the Behavioral risk factor surveillance surveys used to calculate the weighted percentages.

Irrespective of stroke status, nonadherence was highest for annual flu vaccination (Had a stroke: 42.2%, Never had a stroke: 44.0%) and lowest for annual foot exam (Had a stroke 20.4%, Never had a stroke: 22.8%) (Table 2). Except for daily blood glucose check, nonadherence was similar between those who had and had never had a stroke. Following adjustment, people who had suffered a stroke had comparable levels of nonadherence with those who had never had a stroke for annual eye exam (OR: 0.94, 95% CI: 0.82, 1.06), annual foot exam (OR: 0.90, 95% CI: 0.78, 1.04), annual A1C tests (OR: 0.91, 95% CI: 0.80, 1.04), and annual flu vaccination (OR: 0.97, 95% CI: 0.86, 1.08). In contrast, individuals who had a stroke were significantly less likely to be nonadherent with a daily blood glucose check (OR: 0.73, 95% CI: 0.65, 0.82) than those who had never had a stroke.

**Table 2:** 2015–2018 Behavioral Risk Factor Surveillance System study participants nonadherent with American Diabetes Association diabetes management guidelines by stroke status.

**Stroke Status**	**<1 eye exam annually** ^2^		**<1 foot exam annually**		**<1 blood glucose check daily**		**<2 A1C tests annually**		**Did not receive annual flu vaccination**	
	**Weighted % (95% Confidence Interval)**	***P* value**	**Weighted % (95% Confidence Interval)**	***P* value**	**Weighted % (95% Confidence Interval)**	***P* value**	**Weighted % (95% Confidence Interval)**	***P* value**	**Weighted % (95% Confidence Interval)**	***P* value**
Had a stroke	27.4 (26.4, 28.4)	0.232	20.4 (19.4, 21.3)	0.053	30.2 (29.1, 31.3)	<0.001	21.2 (20.3, 22.2)	0.096	42.2 (41.1, 43.4)	0.217
Never had a stroke	29.0 (28.7, 29.3)		22.8 **(**22.5, 23.2**)**		40.1 (39.7, 40.5)		23.0 (22.7, 23.4)		44.0 \(43.6, 44.4)	
**Had a stroke (ref: Never had a stroke)**	**Odds Ratio (Ref: ≥ 1 eye exam annually) (95% Confidence Interval)**	***P* value**	**Odds Ratio (Ref: ≥ 1 foot exam annually) (95% Confidence Interval)**	***P* value**	**Odds Ratio (Ref: ≥ 1 blood glucose check daily) (95% Confidence Interval)**	***P* value**	**Odds Ratio (Ref: ≥ 2 A1C tests annually) (95% Confidence Interval)**	***P* value**	**Odds Ratio (Ref: Did receive annual flu vaccination) (95% Confidence Interval**	***P* value**
	0.94 (0.82, 1.06)	0.313	0.90 (0.78, 1.04)	0.169	0.73 (0.65, 0.82)	<0.001	0.91 (0.80, 1.04)	0.172	0.97 (0.86, 1.08)	0.551

^2^ Survey weighting from the Behavioral risk factor surveillance surveys used to calculate the weighted percentages.

## Discussion

In this national study, we evaluated levels of nonadherence with diabetes care measures for US individuals with diabetes by stroke status. Levels of nonadherence were >20% for all diabetes care measures among stroke survivors. Nonadherence with diabetes care measures was similar by stroke status except for those who had a stroke being less likely to be nonadherent with daily blood glucose check prior to and following adjustment.

Despite evidence that a large proportion of US stroke patients have a history of diabetes, there is relatively little research available on post-stroke diabetes care levels [7]. In a study of 1,460 Medicare patients from 11 US metropolitan regions who were hospitalized for acute ischemic stroke in 2000, Padhi et al. observed that within one year of hospital discharge 47% of patients had not had an annual eye exam and 49% of patients had not had two A1C tests [7]. However, as the Padhi et al. study only includes data on White and Black stroke patients from nearly two decades ago, findings from this study may not be indicative of current diabetes care patterns among US stroke patients with diabetes [7]. Our work expands on the existing post-stroke diabetes care literature by assessing nonadherence with foot exams, blood glucose checks, and flu vaccination in addition to eye exams and A1C tests considered in earlier work as well as demonstrates that nonadherence may have lessened since the early 2000s for some diabetes care measures.

Considerable nonadherence with diabetes care among US stroke survivors could lead to additional hospitalization for diabetes related complications that may develop. Nearly 32% of the 600,000 hospitalizations for diabetes related complications each year are a result of poor diabetes care [18]. Additionally, expenses for these hospitalization total $2.4 billion dollars annually, posing a significant burden to the US healthcare system [18].

Lower nonadherence with diabetes care among stroke survivors may stem from patient, clinician, and caretaking related factors. Individuals who have had stroke could be more aware of the importance of managing their diabetes to prevent further stroke and diabetes related complications than their counterparts who have not had stroke [2]. In addition, doctors may more closely monitor the diabetes care of stroke patients after hospital discharge [2]. Individuals suffering from paralysis, memory loss or other effects of a stroke may also receive additional diabetes care assistance from family members or nursing staff [2]. Further research is needed to better understand the specific roles caregivers and associated social support have in aiding stroke patients to adhere to diabetes care guidelines.

Despite little difference in diabetes care nonadherence by stroke status, additional efforts to improve diabetes care would benefit the large percentage of stroke survivors who continue to be nonadherent with the care guidelines. One such intervention is continued education on diabetes care that is developed specifically for stroke patients and their families [2, 19]. It is also critical that clinicians providing post-stroke care to individuals with diabetes be familiar with the latest ADA care guidelines as a study found that only 67% of US primary care physicians referred their patients to diabetes care education following a diabetes diagnosis [20].

Our study has several limitations that need to be considered. Use of data that consists of self-reported responses such as the BRFSS may result in some misclassification being present in the study [21]. However, there is evidence from both CDC and independent investigator conducted studies that indicates misclassification in BRFSS surveys to be low and that prevalences of diabetes (BRFSS: 9.7%, electronic health records (EHR): 9.4%), obesity (BRFSS: 23.8%, EHR: 22.8%), and hypertension (BRFSS: 29.6%, EHR: 26.3%) from the BRFSS are comparable with information on these measures obtained from the EHR [22, 23]. Although the BRFSS does not include information on the role of caregivers and associated social support, unavailability of these variables is a minor point as the focus of our paper is to determine if there are differences in diabetes care by stroke status and not to identify the factors contributing to these differences [21]. Additionally, there is the potential for some BRFSS survey participants to underreport their nonadherence with diabetes management measures due to a sense of social desirability [24]. If this was the case in the 2015, 2016, 2017, and 2018 BRFSS surveys then actual levels of nonadherence would be even higher than what was found in our study [24].

## Conclusions

In this paper, we examined contemporary post-stroke diabetes care levels in individuals throughout the US. While we found that diabetes care practices are generally comparable between those who have had a stroke and those who had not, there is still considerable nonadherence for multiple measures of diabetes management in stroke survivors. Given that long-term complications arising from stroke can exacerbate diabetes symptoms, additional efforts to reduce nonadherence with post-stroke diabetes care among US stroke patients with diabetes are warranted.
